# Factors affecting posaconazole plasma concentrations: a meta-analysis and systematic review

**DOI:** 10.3389/fphar.2024.1450120

**Published:** 2024-12-19

**Authors:** Ruochen Qu, Yan Liu, Yan Zhao, Ziyi Wang, Shizhao Yuan, Shuai Liu, Jing Yu

**Affiliations:** ^1^ Department of Clinical Pharmacy, The First Hospital of Hebei Medical University, Shijiazhuang, China; ^2^ The Technology Innovation Center for Artificial Intelligence in Clinical Pharmacy of Hebei Province, The First Hospital of Hebei Medical University, Shijiazhuang, China; ^3^ School of Pharmaceutical Sciences, Hebei Medical University, Shijiazhuang, China

**Keywords:** posaconazole oral suspension, posaconazole delayed-release tablets, concentration, plasma, meta-analysis

## Abstract

**Background:**

Posaconazole is a potent antifungal agent widely used to manage invasive fungal infections, especially in immunocompromised individuals. Achieving optimal therapeutic concentrations of posaconazole can be challenging due to interpatient variability, the availability of multiple formulations, and various dosing strategies.

**Methods:**

We conducted a systematic search of PubMed, EMBASE, and the Cochrane Library to identify studies evaluating factors that influence blood concentrations of posaconazole. The primary outcome was the assessment of posaconazole concentrations in relation to various influencing factors, including age, sex, drug interactions, disease state, administered dose, and formulation.

**Results:**

Our analysis included 46 studies involving a total of 8,505 patients. Co-administration of drugs that affect posaconazole metabolism significantly reduced its concentrations. High-fat meals, age, and sex did not have a significant impact on posaconazole oral suspension (POS) concentrations. Diarrhea substantially decreased concentrations of both delayed-release tablets (DRT) and POS. Neither vomiting nor mucositis significantly affected POS concentrations. Acid-suppressing agents, such as H2 receptor antagonists and proton pump inhibitors, notably decreased POS concentrations but had no significant effect on DRT. Comparative studies of different dosage forms revealed significantly higher concentrations with DRT compared to POS.

**Conclusion:**

DRT maintain more stable concentrations than POS and are not affected by acid-suppressing drugs. Given the significant fluctuations in posaconazole concentrations, patients experiencing diarrhea require close monitoring.

**Systematic Review Registration:**

PROSPERO, Identifier CRD42023428822 (https://www.crd.york.ac.uk/prospero/display_record.php?ID=CRD42023428822).

## 1 Introduction

Invasive fungal infections (IFIs) pose significant challenges in clinical practice, particularly among immunocompromised patients, such as those undergoing hematopoietic stem cell transplantation (HSCT), solid organ transplantation (SOT), or those suffering from hematologic malignancies or HIV/AIDS (von Lilienfeld-Toal et al., 2019). Posaconazole, a second-generation triazole antifungal agent, exhibits broad-spectrum activity against various clinically relevant fungal pathogens, including Aspergillus spp., *Candida* spp., and Zygomycetes (Chen et al., 2020). Its efficacy and favorable safety profile have led to its widespread use in the prophylaxis and treatment of IFIs (Van Daele et al., 2020). Research has shown a correlation between low posaconazole concentrations and the occurrence of breakthrough invasive fungal infections (bIFIs) (Dolton et al., 2012). Recommended concentrations exceed 700 ng/mL for prophylaxis and 1,000 ng/mL for treatment (Kably et al., 2022; McCreary et al., 2023; Gómez-López, 2020). Additionally, a meta-analysis suggests that a concentration of 500 ng/mL is effective for prevention, while the toxicity threshold for trough concentrations is set at 3,750 ng/mL (Chen et al., 2018).

Achieving optimal posaconazole exposure remains challenging due to significant interpatient variability in pharmacokinetics, which arises from individual differences in drug absorption, distribution, metabolism, and elimination. Posaconazole is available in several formulations, including posaconazole oral suspension (POS), delayed-release tablets (DRT), and intravenous solutions, each with distinct pharmacokinetic characteristics. Patient-specific factors, such as age, concomitant medications, renal and hepatic function, and underlying disease conditions, can significantly influence posaconazole exposure.

## 2 Aim

This meta-analysis and systematic review aimed to investigate factors influencing posaconazole concentrations and to provide insights for optimizing antifungal therapy in clinical practice.

## 3 Methods

This systematic review and meta-analysis was conducted in accordance with the Preferred Reporting Items for Systematic Reviews and Meta-Analyses (PRISMA) guidelines. The review was registered with PROSPERO (registration number CRD42023428822). Quantitative data synthesis was performed using meta-analytic techniques. Analyses of posaconazole concentrations considered various formulations, dosing regimens, renal function, concomitant medications, and patient populations.

### 3.1 Search strategy and screening

We conducted a comprehensive search of articles published before 29 December 2023, in three electronic databases: the Cochrane Library, EMBASE, and PubMed. The search strategy was developed using MeSH/EMTREE terms and free-text keywords to target relevant populations, outcomes, and study types. The following search terms in the PubMed were used in the search queries: ((((((“Plasma” [MeSH]) OR (plasma [Title/Abstract])) OR (“Blood” [MeSH]) OR (blood [Title/Abstract])) OR (“Serum” [MeSH]) OR (serum [Title/Abstract])) OR ((“Drug Monitoring” [MeSH]) OR (“Monitoring, Drug” [Title/Abstract])) OR (“therapeutic drug monitoring” [Title/Abstract])) OR (concentration [Title/Abstract])) AND ((“posaconazole” [Supplementary Concept]) OR (“Noxafil” [Title/Abstract]) OR (posaconazole [Title/Abstract])).

Two methodologically trained reviewers independently screened the titles and abstracts to determine whether the articles met the inclusion criteria. Discrepancies were resolved through consensus or, when necessary, arbitration by a third reviewer. Full-text articles were then reviewed, and relevant data were extracted. The reasons for inclusion or exclusion were documented. Studies published in non-English languages, case reports, letters, and meeting minutes were excluded.

### 3.2 Inclusion criteria

The inclusion criteria were studies involving patients or healthy volunteers using posaconazole. Studies without available concentration data were excluded. We included randomized controlled trials (RCTs) that assigned patients to groups based on different influencing factors, as well as observational studies, prospective cohorts, retrospective cohorts, case-control studies, and intervention studies. We excluded case reports, comments, editorials, reviews, studies lacking concentration data, studies that did not investigate factors affecting concentration levels, and studies that lacked a control group.

### 3.3 Study selection and data abstraction

Two reviewers independently screened the titles and abstracts of all studies, and they retrieved through the search strategies based on the predefined inclusion criteria. The following information was extracted: (a) publication details, including authors, year of publication, and country of study; (b) study design, specifying whether it was an RCT or an observational study; (c) patient demographics, including the number of participants, their ages, and genders; (d) diagnosis, dose administered, frequency of administration, and route of administration; (e) posaconazole concentrations, including means, medians, ranges, and interquartile ranges. Discrepancies in data extraction were resolved through discussion.

### 3.4 Assessment of study quality

The quality of the studies was assessed using the Newcastle-Ottawa Scale (NOS). Each study could receive a maximum of nine stars, with one star awarded per item, except for comparability, which could receive up to two stars. Studies scoring 0–3 stars were considered to have a high risk of bias, 4–6 stars indicated a moderate risk, and 7–9 stars suggested a low risk of bias.

### 3.5 Outcome measure

This review evaluated the impact of various factors, including age, sex, drug interactions, disease states, administered doses, and formulations, on posaconazole blood concentrations.

### 3.6 Statistical analyses

Data were analyzed using Review Manager version 5.4 (Cochrane Collaboration, Oxford, England). Continuous outcomes were measured by mean difference (MD) and reported with 95% confidence intervals (CIs). Results were presented descriptively for outcomes that were unsuitable for pooled effect estimates. For studies providing only individual patient data, the mean ± standard deviation was calculated. If studies did not directly report means and standard deviations, these were estimated using formulas from previous studies (Luo et al., 2018; Shi et al., 2020). Cochran’s Q test and I^2^ statistics were used to assess statistical heterogeneity and inconsistent treatment effects across studies. If there was no significant heterogeneity between studies, we analyzed using a fixed-effects model and vice versa using a random-effects model.

## 4 Results

### 4.1 Study characteristics and quality assessment

**FIGURE 1:**
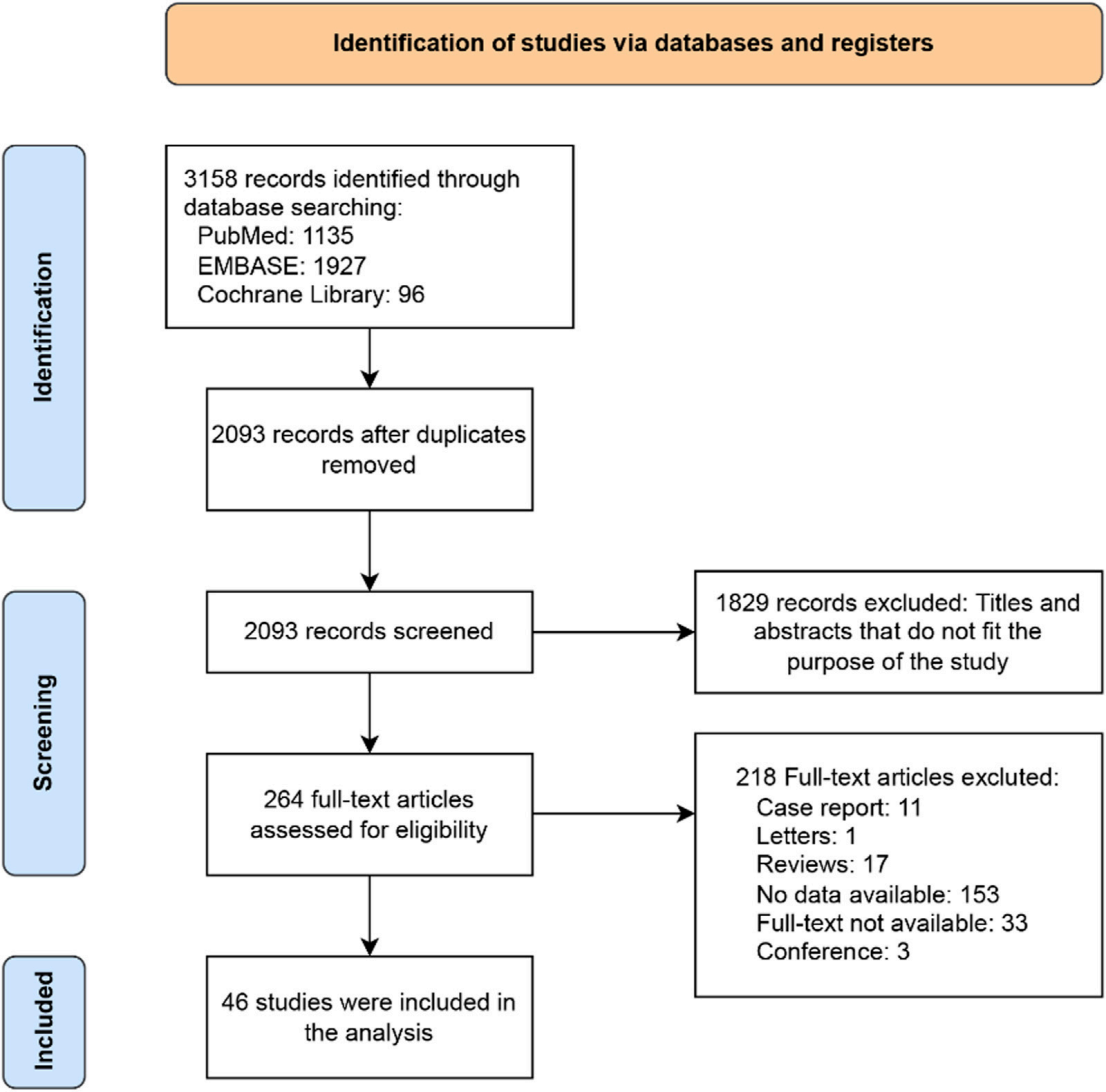
Study flowchart.

A total of 46 studies published between 2007 and 2020 met the inclusion criteria and were included in the analysis. The study selection process isdepicted in Figure 1. These studies had 8,505 patients, with individual study sample sizes ranging from 2 to 513 (Table 1). The patient populations were diverse, covering various indications for both prophylaxis and treatment of invasive fungal infections. Among the included studies, 8 were RCTs, 20 were retrospective, 7 were prospective, and 11 were parallel-group studies. Data on posaconazole concentrations in patients included in the quantitative analysis are detailed in Supplemental Table 1.

**TABLE 1:** Characteristics of the included studies.

Study	Design of study	Country of study	Age of patients	Gender (male/female)	Population	Purpose	Formulation	SD/MD	Dosage/d	PK	Interventions			
											Test group (n)	Control group(n)	Endpoints	NOS
Gubbins et al. (2006)	Nonrandomized,single-center, open-label, parallel-group	America	Mean: 52.5 ± 9.4	NA	Neutropenic patients undergoing high-dose chemotherapy and stem cell transplantation	Treatment	POS	MD	_	C_max_ (ng/ml)	400 mg QD (n = 14)	200 mg QID (n = 7)	Dosage	4
Krishna et al. (2007a)	Randomized controlled trial	America	Mean: 36 (20-45)	36/0	Healthy men	_	TAB	MD	200 mg	C_max_ (ng/ml)	PCZ (200 mg QD) with phenytoin (200 mg QD) (n = 36)	PCZ alone (200 mg QD) (n = 36)	DDI	5
Courtney et al. (2005)	Open-label, parallel-group	America	52.5 ± 15.5	17/7	Healthy subjects and in those with mild [CL (CR) = 50–80 mL/min], moderate [CL(CR) = 20–49 mL/min]	_	POS	SD	400 mg	C_max_ (ng/ml)	Mild (n = 6) Moderate (n = 6)	Healthy Subjects (n = 6)	Degrees of Chronic Renal Disease	3
Sansone-Parsons et al. (2006)	Open-label, single-center, randomized study	America	18–55	12/12	Healthy subjects	_	POS	SD	400 mg	C_max_ (ng/ml)	PCZ with Boost Plus (n = 24)	PCZ alone (n = 24)	Nutritional supplement	4
Krishna et al. (2007b)	Multicenter, open-label study	America	NA	136/70	Patients who were intolerant of or had invasive fungal infection refractory to standard antifungal therapies	Treatment	POS	MD	200 mg QID or 400 mg BID (800 mg)	C_av_ (ng/ml)	Juvenile (<18) (n = 12)	Adult (18–64) (n = 194)	Age	5
Sansone-Parsons et al. (2007)	Randomized, placebo-controlled, blinded study, open-label, parallel-group study	America	NA	NA	Healthy adult subjects	_	POS	MD	800 mg	C_max_ (ng/ml)	Young (18–45) (n = 24)	Elderly (≥65) (n = 24)	Age	5
Krishna et al. (2007c)	Non-randomized, open-label, parallel-group, multiple-dose	America	Mean: 27 (range, 20–40)	20/0	Healthy men	_	TAB	MD	200 mg	C_max_ (ng/ml)	PCZ (200 mg QD) with rifabutin (200 mg QD) (n = 8)	PCZ alone (200 mg QD) (n = 12)	DDI	4
Krishna et al. (2007d)	Multicenter,randomized, double-blind, double_x005f dummy, parallel-group trial	America	NA	165/76	Prophylactic posaconazole users without invasive fungal infection	Prophylaxis	POS	MD	600 mg	C_av_ (ng/ml)	Female (n = 76)	Male (n = 165)	Gender	6
											18–45 (n = 133)	>45 (n = 106)	Age	
											Acute (n = 158)	Chronic (n = 82)	GVHD status	
											Present (n = 18)	Absent (n = 223)	Diarrhea	
Krishna et al. (2008)	Prospective, randomized, multicenter, evaluator-blinded trial	America	NA	111/83	Patients who have neutropenia with an absolute neutrophil count of 500 cells/mm^3^ or less, lasting for 7 days or more	Prophylaxis	POS	MD	600 mg	C_av_ (ng/ml)	Female (n = 83)	Male (n = 111)	Gender	4
											13–18 (n = 7)	18–45 (n = 61) 45–65 (n = 88) >65 (n = 38)	Age	
											≥ 2 ULN (n = 32)	< 2 ULN (n = 149)	γ-Glutamyl transferase level	
											≥ 2 ULN (n = 30)	< 2 ULN (n = 163)	Liver enzyme levels	
											Mild to moderate (n = 55) Severe to life threatening (n = 2)	None (n = 137)	Diarrhea	
											Mild to moderate (n = 19)	None (n = 174)	Vomiting	
											Yes (n = 61)	No (n = 133)	H_2_-receptor antagonist	
											Yes (n = 86)	No (n = 108)	Proton pump inhibitor	
											Grades 1–2 (n = 66) Grades 3–4 (n = 3)	No (n = 123)	Mucositis	
Lebeaux et al. (2009)	Monocentric retrospective study	France	48.7 ± 15	38/16	Adult patients whose PPC were measured after at least 5 days of PSZ therapy	Prophylaxis	Oral formulation	MD	600 mg	NA	Yes (n = 14)	No (n = 22)	Diarrhea	3
											Yes (n = 6)	No (n = 30)	Mucositis	
						Treatment	Oral formulation		800 mg		Yes (n = 4)	No (n = 14)	Diarrhea	
											Yes (n = 6)	No (n = 12)	digestive diseases	
Moton et al. (2010)	Open-label, parallel-group, single center study	America	18–75	23/14	19 with hepatic impairment and 18 healthy subjects	_	POS	SD	400 mg	C_max_ (ng/ml)	Mild (n = 6)	Normal (n = 6)	Hepatic impairment (Child-Pugh scoring system)	3
											Moderate (n = 6)	Normal (n = 6)		
											Severe (n = 6)	Normal (n = 6)		
Krishna et al. (2011)	Randomized, parallel-group, multicenter, investigator-blinded study	America	NA	NA	Patients who had a clinical and mycologic diagnosis of onychomycosis	Treatment	Oral posaconazole	MD	_	C_min_ (ng/ml)	400 mg QD (n = 30)	200 mg QD (n = 33)	Dosage	4
Bryant et al. (2011)	Retrospective study	America	54.1 ± 17.8	11/10	Patients with acute myelogenous leukaemia or myelodysplastic syndrome	Prophylaxis	POS	MD	600 mg	C_ss_ (μg/mL)	Yes (n = 5)	No (n = 16)	Diarrhea	3
											Yes (n = 5)	No (n = 16)	Vomiting	
											Yes (n = 2)	No (n = 19)	Mucositis	
											Yes (n = 19)	No (n = 2)	PPI or H_2_ antagonist	
											Female (n = 10)	Male (n = 11)	Gender	
Ray et al. (2011)	RCT	Australia	NA	19/8	Patients in the general intensive care unit	Prophylaxis	POS: via the NG tube	MD	800 mg	Mean C_min_ steady-state (ng/ml)	400 mg bid (n = 13)	200 mg qid (n = 14)	Dosage	5
Krishna et al. (2012)	Single-centre, randomized(according to a computer-generated sponsor-provided randomization code), placebo-controlled study	America	mean: 45.9 (range, 31–59)	11/8	Healthy subjects	_	TAB	SD	_	C_max_ (ng/ml)	400 mg (n = 9)	200 mg (n = 10)	Dosage	5
								MD			400 mg (n = 8)	200 mg (n = 8)		
Tonini et al. (2012)	Retrospective, observational study	France	48.6 ± 10.8	18/11	Patient population was limited to recipients of HSCT who developed GVHD	Prophylaxis	POS	MD	600 mg	C_min_ (mg/L)	GVHD: GI (n = 14)	GVHD: non-GI (n = 15)	Gastrointestinal (GI) GVHD	4
Ross et al. (2012)	Observational study	America	—	—	Haematological malignancy patients	Prophylaxis	POS	MD	—	Css (μg/mL)	400 mg bid (n = 34)	200 mg tid (n = 20)	Dosage	4
Crombag et al. (2012)	Retrospective analysis	Netherlands	44.7 (19-64)	11/6	Hematology patients	Prophylaxis and treatment	POS	MD	600 mg and 800 mg	NA	Yes (n = 12)	No (n = 5)	PPI	4
Bernardo et al. (2013)	Retrospective clinical study	America	Median: 11.5 (range: 0.5-23.2)	18/15	Patients with cancer who received posaconazole for the treatment of suspected or proven infections	Treatment	POS	MD	Patients weighing less than 34 kg are 18–24 mg/kg daily. Patients aged 13 years or older or those weighing 34 kg or more take 800 mg daily.	Css (μg/mL)	<13 years (n = 21)	≥13 years (n = 12)	Age	4
Cojutti et al. (2013)	Retrospective, observational study	Italy	—	10/14	Patients with acute myeloid leukemia who underwent antifungal prophylaxis with posaconazole	Prophylaxis	POS	MD	600	Cmin (mg/L)	Yes (n = 11)	No (n = 10)	PPI	4
				9/12					—		200 mg q6h (n = 10)	200 mg q8h (n = 11)	Dosage	
Heinz et al. (2013)	Retrospective analysis	Germany	Median: 53 (range: 20–73)	35/29	Receiving posaconazole after allogeneic stem cell recipients	—	POS	MD	—	Css (ng/mL)	400 mg bid (n = 56)	200 mg tid (n = 12)	Dosage	4
Maertens et al. (2014)	Open-label,multicenter study	Germany	49.1 ± 14.7 and 52.4 ± 13.4	26/19	Patients at high risk for invasive fungal disease	Prophylaxis	IV	MD	—	Cavg (ng/mL)	300 mg qd (n = 24)	200 mg qd (n = 21)	Dosage	4
Kersemaekers et al. (2015)	A single-center, 2-part, randomized, placebocontrolled, third-party blind, rising single- and multiple-dose study	America	Range: 18-56	—	Healthy subjects	—	IV	SD	—	Cmax (ng/ml)	300 mg (n = 9)	200 mg (n = 9)	Dosage	6
Durani et al. (2015)	Retrospective analysis	America	—	—	patients taking DR posaconazole tablets	Prophylaxis and treatment	POS and DRT	MD	—	Css (ng/mL)	DRT (n = 32)	POS (n = 61)	Formulation	4
Cumpston et al. (2015)	Retrospective analysis	America	—	76/74	Acute myelogenous leukemia (AML) or high-grade myelodysplastic syndrome (MDS) who were admitted to the inpatient hematologic malignancy service	Prophylaxis	POS and DRT	MD	POS: 600–800 mg/Day; DRT: 300 mg/Day	Css (ng/mL)	DRT (n = 32)	POS (n = 118)	Formulation	4
Miceli et al. (2015)	Single centre retrospective analysis	America	Mean: 53 (range: 19–77)	16/12	Patients undergoing chemotherapy for AML and HCT recipients who received delayed release posaconazole tablets	Prophylaxis	DRT	MD	300 mg (300 mg twice a day on the first day)	Css (μg/mL)	Yes (n = 5)	No (n = 23)	Diarrhoea	4
											Yes (n = 23)	No (n = 5)	PPI/H2RA	
											≥ 90 (n = 6)	< 90 (n = 22)	Body weight (kg)	
											≥ 30 (n = 7)	< 30 (n = 21)	BMI	
Vanstraelen et al. (2016)	Prospective study	Belgium	—	47/33	Allogeneic HSCT patients receiving posaconazole prophylaxis	Prophylaxis	POS	MD	600 mg	Cmin (mg/L)	HSCT patients (n = 34)	no-HSCT patients (n = 33)	HSCT	4
Cornely et al. (2016)	Open-label, multicentre study	Germany	51.0 ± 14.1	—	Patients at high risk for IFD	Prophylaxis	DRT	MD	300 mg (300 mg twice a day on the first day)	Cmin (ng/mL)	HSCT (n = 79)	AML/MDS (n = 107)	Disease state	4
Heinz et al. (2016)	Single-center analysis	Germany	—	9/18	Pediatric patients under 17 years of age with hemato-oncological malignancies	Prophylaxis	POS	MD	4 mg/kg three times a day	Cmin (ng/mL)	Higher-Fat Nutrition (n = 10)	Regular Nutrition (n = 17)	Different nutrition regimens	4
Pham et al. (2016)	Retrospective single-centre cohort study	America	—	161/101	Adult haematological cancer patients (≥18 years) initiated on PTF or OSF	Prophylaxis and treatment	PTF or OSF	MD	FDA-approved dosing of posaconazole	Cmin (μg/mL)	Posaconazole tablet formulation (n = 6)	oral suspension formulation (n = 176)	Posaconazole tablet formulation (PTF) and oral suspension formulation (OSF)	4
											omeprazole (n = 40)	No acid suppression (n = 34)	Tablet	
											omeprazole (n = 9)	No acid suppression (n = 67)	Suspension	
											famotidine (n = 12)	No acid suppression (n = 34)	Tablet	
											famotidine (n = 100)	No acid suppression (n = 67)	Suspension	
Suh et al. (2017)	Prospective study	South Korea	—	119/95	Received posaconazole as a prophylactic antifungal agent	Prophylaxis	POS and tablet	MD	POS :200 mg tid; Tablet: 300mg qd	Cmean (ng/mL)	posaconazole tablet (n = 40)	POS (n = 174)	Formulation	4
Morgan Belling, et al (2017)	Retrospective analysis	America	—	96/86	Patients with acute myeloid leukemia or myelodysplastic syndromes and using posaconazole to prevent fungal infections	Prophylaxis	POS and tablet	MD	POS :600-800 mg; Delayed-release tablet: 200-300 mg	Css (ng/mL)	Delayed-release tablet (n = 64)	POS (n = 118)	Formulation	4
Stelzer et al. (2017)	Retrospective analysis	Germany	—	—	Adult lung transplant recipients	Therapy	POS and tablet	MD	POS :800 mg; Tablet: 300 mg	Css (ng/mL)	Posaconazole tablets (n = 64)	POS (n = 64)	Formulation	4
Peterlin et al. (2018)	Prospective monocentric noninterventional study	France	—	—	Patients aged 18 years or over who received GR-posa prophylactically	Prophylaxis	Gastro-resistant posaconazole tablet	MD	300 mg (300 mg twice a day on the first day)	Cmin (ng/mL)	Graft-versus-host disease after allogeneic hematopoietic stem cells transplantation (n = 19)	Induction chemotherapy (n = 24)	HSCT	4
Suh et al. (2018)	Prospectively study	South Korea	53.9 ± 13.1	—	Patients aged 18 years old who underwent chemotherapy and who were treated with aPOS at 200 mg three times a day as a prophylactic antifungal agent		POS	MD	600 mg	—	TT (n = 94)	GT (n = 36)	gene	4
Launay et al. (2018)	Prospective study	France	—	—	Lung transplant patients	Prophylaxis and treatment	Delayed-release oral tablet	MD	300 mg (300 mg twice a day on the first day)	Cmin(μg/mL)	PPI (n = 19)	Without PPI (n = 6)	PPI	4
Kozuch et al. (2018)	Two-center retrospective cohort study	America	56 ± 13.7	—	Lung transplant patients who received posaconazole delayed release tablets	Prophylaxis	Delayed Release Tablets	MD	—	Cmin(μg/mL)	400 mg (n = 20)	200 mg (n = 19)	Dosage	4
Jeong et al. (2018)	A single-centre, retrospective observational study	Australia	—	83/42	Lung transplant recipients	Prophylaxis and treatment	POS and modified release tablets	MD	POS: 800 mg/Day; DRT: 300 mg/Day	Cmin(mg/L)	Tab (n = 78)	POS (n = 47)	Formulation	4
Liebenstein et al. (2018)	Retrospective case-control study	America	—	45/29	Adult inpatients with acute myeloid leukemia undergoing chemotherapy, who received posaconazole for invasive fungal infection	Prophylaxis	Delayed-release tablet and POS	MD	POS: 600 mg/Day; DRT: 300 mg/Day	Css (ng/mL)	Tab (n = 40)	POS (n = 30)	Formulation	4
Stelzer et al. (2018)	Retrospective, observational study longitudinally	Germany	—	10/14	Lung transplantation-recipients	prophylaxis	Delayed-release tablet and POS	MD	POS: 600 mg/Day; DRT: 300 mg/Day	Cmin(mg/L)	Tab (n = 9)	POS (n = 9)	Formulation	4
						treatment			POS: 800 mg/Day; DRT: 300 mg/Day		Tab (n = 15)	POS (n = 15)		
Leclerc et al. (2018)	Observational, single-centre study	France	53.7 ± 13.5	—	Patients with haematologic malignancies who were treated with PCZ for antifungal	prophylaxis	Delayed-release tablet and POS	MD	POS: 627 ± 143 mg/Day; DRT: 290 ± 45 mg/Day	Cmin(mg/L)	Tab (n = 50)	POS (n = 104)	Formulation	4
							Delayed-release tablet				YES (n = 6)	NO (n = 44)	Diarrhoea	
Gautier-Veyret et al. (2019)	Retrospective study	France	53.0 (22.0–64.7)	41/36	Adult allogeneic hematopoietic stem-cell transplant patients with graft-versus-host disease	prophylaxis	Delayed-release tablet and POS	MD	POS: 600 (600–800) mg/Day; DRT:300 (200–300) mg/Day	Cmin (mg/L)	Tab (n = 41)	POS (n = 29)	Formulation	4
Li et al. (2020)	Open, prospective, observational single-center study	China	32.7 ± 13.8	48/26	Hematology patients ≥13 years old, who underwent HSCT transplantation or induction chemotherapy	prophylaxis	POS	MD	600mg/day	Cmin (ng/mL)	YES (n = 53)	NO (n = 21)	PPI	3
Lai et al. (2020)	Retrospective, single-centre study	Australia	Median 5 (range: 33 months-12 years)	39/31	Immunocompromised children <13 years	prophylaxis	POS	MD	Starting dose of 5 mg/kg every 8 h for 7 days	Cmin(ng/mL)	YES (n = 14)	NO (n = 56)	PPI	4
											YES (n = 18)	NO (n = 52)	metoclopramide	
											YES (n = 6)	NO (n = 64)	mucositis	
											YES (n = 2)	NO (n = 68)	ranitidine	
											YES (n = 16)	NO (n = 54)	enteral feeding	
											YES (n = 19)	NO (n = 51)	HSCT	
Oh et al. (2020)	Retrospective study	Korea	—	152/90	Adult patients with hematologic malignancies	prophylaxis	Delayed-release tablet and POS	MD	Tab: 300 mg/day (300 mg twice a day on the first day) POS: 600 mg/day	Css (μg/mL)	Tab (n = 154)	POS (n = 8)	Formulation	4
Chae et al. (2020)	Retrospective single-center analysis	Korea	—	330/305	Aged 18 years or older	prophylaxis	Delayed-release tablet and POS	MD	Tab: 300 mg/day (300 mg twice a day on the first day) POS: 600 mg/day	Css (μg/mL)	Tab (n = 513)	POS (n = 122)	Formulation	4
			Median 48 (IQR: 37-57)	261/252			Delayed-release tablet	MD	Tab: 300 mg/day (300 mg twice a day on the first day)		HSCT with GVHD group (n = 174)	remission induction group (n = 339)	GVHD	

NA, not available; PCZ, posaconazole; POS, posaconazole oral suspension; DRT, Delayed-release tablet; SD, single-dose; MD, multiple-dose; DDI, drug-drug interactions; Cavg, average concentration at steady state; Css, steady-state concentration; RCT, randomized controlled trials; GVHD, graft-versus-host disease; HSCT, haematopoietic stem cell transplantation.

### 4.2 Drug-drug interaction

**FIGURE 2:**
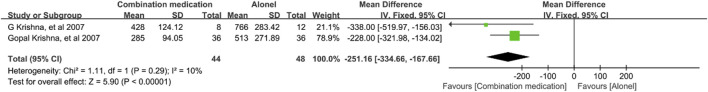
Forest plot of posaconazole concentrations for different combinations of medications.

Two studies (Krishna et al., 2007c; Krishna et al., 2007b) involving 92 patients evaluated the impact of concurrent medication use on posaconazole concentrations in DRT form. The results indicated that the combined use of rifabutin and phenytoin significantly reduced drug blood levels in healthy volunteers [mean difference [MD] −251.16 ng/mL; 95% confidence interval [CI], −334.66 to −167.66; *p* < 0.001; Figure 2].

### 4.3 Nutrition regimens

**FIGURE 3:**
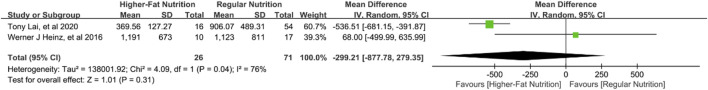
Forest plot of the effect of high-fat nutrition on the concentration of posaconazole oral suspension.

Three studies (Heinz et al., 2016; Lai et al., 2020; Sansone-Parsons et al., 2006) involving 145 patients assessed the effect of high-fat nutrition on POS concentrations. Quantitative analysis from two of these studies (Heinz et al., 2016; Lai et al., 2020) found no significant impact of high-fat diets on drug concentrations [mean difference [MD] −299.21 ng/mL; 95% confidence interval [CI], −877.78 to 279.35; *p* = 0.310; Figure 3]. However, in healthy volunteers, a single 400 mg dose of POS taken with a nutritional supplement resulted in a 3.4-fold increase in the maximum serum concentration of posaconazole from 0 to 72 h (Sansone-Parsons et al., 2006).

### 4.4 Age

**FIGURE 4:**
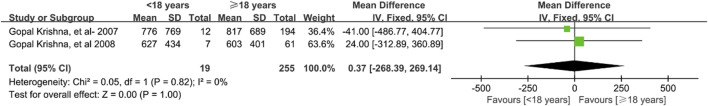
Forest plot of the effect of age 18 years up and down on the concentration of posaconazole oral suspension.

**FIGURE 5:**
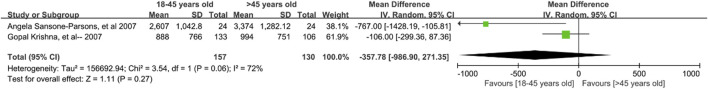
Forest plot of the effect of age 45 years up and down on the concentration of posaconazole oral suspension.

Five studies (Krishna et al., 2007d; Krishna et al., 2008; Sansone-Parsons et al., 2007; Krishna et al., 2007a; Bernardo et al., 2013) involving 594 patients explored the effect of patient age on posaconazole concentrations. Quantitative analysis of four studies (Krishna et al., 2007d; Krishna et al., 2008; Sansone-Parsons et al., 2007; Krishna et al., 2007a) found no significant differences in concentrations between patients younger and older than 18 years [mean difference [MD] 0.37 ng/mL; 95% confidence interval [CI], −268.39 to 269.14; *p* = 1.000; Figure 4) or between those younger and older than 45 years (MD −357.78 ng/mL; 95% CI, −986.90 to 271.35; *p* = 0.270; Figure 5]. Higher blood levels were observed in pediatric patients under 13 years of age who were dosed based on body weight (Bernardo et al., 2013).

### 4.5 Sex

Three studies (Krishna et al., 2008; Krishna et al., 2007a; Bryant et al., 2011) involving 456 patients investigated the effect of gender on posaconazole concentrations. The results revealed no significant differences between male and female patients [mean difference [MD] −5.77 ng/mL; 95% confidence interval [CI], −76.57 to 88.11; *p* = 0.890; Supplemental Figure S1].

### 4.6 Diarrhea

Six studies (Krishna et al., 2008; Krishna et al., 2007a; Bryant et al., 2011; Lebeaux et al., 2009; Miceli et al., 2015; Leclerc et al., 2018) involving 568 patients assessed the impact of diarrhea on posaconazole concentrations. Analysis of four studies (Krishna et al., 2008; Krishna et al., 2007a; Bryant et al., 2011; Lebeaux et al., 2009) on POS and two (Miceli et al., 2015; Leclerc et al., 2018) on DRT showed that diarrhea significantly reduced drug concentrations regardless of formulation [POS: mean difference [MD] −252.14 ng/mL; 95% confidence interval [CI], −332.26 to −172.02, *p* < 0.001; Supplemental Figure S2; DRT: MD -670.27 ng/mL; 95% CI, −756.86 to −583.67, *p* < 0.001; Supplemental Figure S3].

### 4.7 Vomiting

Two studies (Krishna et al., 2008; Bryant et al., 2011) involving 214 patients explored the impact of vomiting on posaconazole concentrations. The analysis found no significant effect [mean difference [MD] −15.43 ng/mL; 95% confidence interval [CI], −148.76 to 117.90; *p* = 0.820; Supplemental Figure S4].

### 4.8 H2-receptor antagonist

Five studies (Lai et al., 2020; Krishna et al., 2008; Bryant et al., 2011; Miceli et al., 2015; Pham et al., 2016) involving 526 patients examined the effect of H2 receptor antagonists (H2A) on posaconazole concentrations. Four studies (Lai et al., 2020; Krishna et al., 2008; Bryant et al., 2011; Miceli et al., 2015; Pham et al., 2016) focused on POS, while two (Miceli et al., 2015; Pham et al., 2016) focused on DRT. The results indicated that co-administration of H2A did not significantly affect DRT concentrations [mean difference [MD] −285.74 ng/mL; 95% confidence interval [CI], −847.06 to 275.58; *p* = 0.320; Supplemental Figure S5]. However, H2A significantly reduced POS concentrations (MD -197.83 ng/mL; 95% CI, −377.64 to −18.02; *p* = 0.030; Supplemental Figure S6).

### 4.9 Proton pump inhibitor

Nine studies (Lai et al., 2020; Krishna et al., 2008; Bryant et al., 2011; Miceli et al., 2015; Pham et al., 2016; Crombag et al., 2012; Cojutti et al., 2013; Launay et al., 2018; Li et al., 2020) involving 600 patients investigated the impact of proton pump inhibitors (PPIs) on posaconazole concentrations. Three studies (Miceli et al., 2015; Pham et al., 2016; Launay et al., 2018) focused on DRTs, while seven (Lai et al., 2020; Krishna et al., 2008; Bryant et al., 2011; Pham et al., 2016; Crombag et al., 2012; Cojutti et al., 2013; Li et al., 2020) focused on POS. The findings showed that co-administration of PPIs did not significantly affect DRT concentrations [mean difference [MD] −261.65 ng/mL; 95% confidence interval [CI], −638.21 to 114.92; *p* = 0.170; Supplemental Figure S7], but significantly reduced POS concentrations (MD -179.99 ng/mL; 95% CI, −246.83 to −113.14; *p* < 0.001; Supplemental Figure S8).

### 4.10 Mucositis

Nine studies (Lai et al., 2020; Krishna et al., 2008; Bryant et al., 2011; Miceli et al., 2015; Pham et al., 2016; Crombag et al., 2012; Cojutti et al., 2013; Launay et al., 2018; Li et al., 2020) involving 600 patients investigated the impact of proton pump inhibitors (PPIs) on posaconazole concentrations. Three studies (Miceli et al., 2015; Pham et al., 2016; Launay et al., 2018) focused on DRTs, while seven (Lai et al., 2020; Krishna et al., 2008; Bryant et al., 2011; Pham et al., 2016; Crombag et al., 2012; Cojutti et al., 2013; Li et al., 2020) focused on POS. The findings showed that co-administration of PPIs did not significantly affect DRT concentrations [mean difference [MD] −261.65 ng/mL; 95% confidence interval [CI], −638.21 to 114.92; *p* = 0.170; Supplemental Figure S7], but significantly reduced POS concentrations (MD -179.99 ng/mL; 95% CI, −246.83 to −113.14; *p* < 0.001; Supplemental Figure S8).

### 4.11 Formulation differences

Thirteen studies (Leclerc et al., 2018; Pham et al., 2016; Durani et al., 2015; Cumpston et al., 2015; Suh et al., 2017; AuthorAnonymous et al., 2017; Stelzer et al., 2017; Jeong et al., 2018; Liebenstein et al., 2018; Stelzer et al., 2018; Gautier-Veyret et al., 2019; Oh et al., 2020; Chae et al., 2020) involving 2,343 patients assessed differences in posaconazole concentrations between the oral suspension (POS) and DRT formulations. The analysis revealed significantly higher blood concentrations in patients using DRTs compared to those using POS [mean difference [MD] 845.86 ng/mL; 95% confidence interval [CI], 675.10 to 1,016.63; *p* < 0.001; Supplemental Figure S10].

### 4.12 Hematopoietic stem cell transplantation

Four studies (Lai et al., 2020; Chae et al., 2020; Vanstraelen et al., 2016; Peterlin et al., 2018) involving 693 patients explored the effects of hematopoietic stem cell transplantation (HSCT) on posaconazole concentrations. Two studies (Chae et al., 2020; Peterlin et al., 2018) specifically analyzed concentrations in patients undergoing HSCT and induction chemotherapy with dDRTs, finding no significant differences [mean difference [MD] 601.77 ng/mL; 95% confidence interval [CI], −355.53 to 1,559.08; *p* = 0.220; Supplemental Figure S11]. Additional findings from Lai et al. (2020) and Li et al. (2020) indicated lower plasma concentrations in hematologic patients receiving HSCT with POS compared to those not undergoing HSCT (288.46 ng/mL vs. 1,144.06 ng/mL). Similarly, pediatric patients under 13 years of age who received HSCT had lower posaconazole concentrations than those who did not undergo HSCT (569.11 ng/mL vs. 863.29 ng/mL) (Lai et al., 2020).

### 4.13 Dosage

Ten studies (Cojutti et al., 2013; Gubbins et al., 2006; Krishna et al., 2011; Ray et al., 2011; Krishna et al., 2012; Ross et al., 2012; Heinz et al., 2013; Maertens et al., 2014; Kersemaekers et al., 2015; Kozuch et al., 2018) involving 372 patients assessed posaconazole concentrations across different formulations, including oral suspension (POS),DRT, and intravenous (IV) administration, at varying doses. For POS, five studies (Cojutti et al., 2013; Gubbins et al., 2006; Krishna et al., 2011; Ross et al., 2012; Heinz et al., 2013) found no significant differences in concentrations between daily doses of 200 mg and 400 mg [mean difference [MD] 3.24 ng/mL; 95% confidence interval [CI], −267.94 to 274.42; *p* = 0.980; Supplemental Figure S12] or between 600 mg and 800 mg (MD 152.64 ng/mL; 95% CI, −182.17 to 487.46; *p* = 0.370; Supplemental Figure S13). For DRTs, two studies (Krishna et al., 2012; Kozuch et al., 2018) showed significantly higher concentrations with a daily dose of 400 mg compared to 200 mg (MD 880.15 ng/mL; 95% CI, 266.65 to 1,493.64; *p* = 0.005; Supplemental Figure S14). For intravenous administration, two studies (Maertens et al., 2014; Kersemaekers et al., 2015) demonstrated that a daily dose of 300 mg resulted in significantly higher concentrations than 200 mg (MD 318.48 ng/mL; 95% CI, 3.82 to 633.15; *p* = 0.050; Supplemental Figure S15). In critically ill patients administered posaconazole via nasogastric tube, concentrations remained low with both 400 mg twice-daily and 200 mg four-times-daily regimens (Ray et al., 2011).

### 4.14 Other concentration influencing factors

#### 4.14.1 Metoclopramide

A study by Lai et al. (2020) reported that co-administration of the gastric stimulant metoclopramide decreased posaconazole concentrations in pediatric patients under 12 years of age receiving POS prophylactically (500.11 ng/mL vs. 887.52 ng/mL).

#### 4.14.2 Renal and hepatic function

A study by Courtney et al. (2005) comparing healthy volunteers with patients experiencing renal impairment found that posaconazole concentrations were not affected by hemodialysis, suggesting that renal disease severity does not necessitate dosage adjustments. Another study by Moton et al. (2010) found no significant effect of varying degrees of hepatic impairment on posaconazole concentrations. The influence of elevated gamma-glutamyl transferase (γ-GT) levels was also considered clinically insignificant (Krishna et al., 2008).

#### 4.14.3 Digestive system diseases

In patients with digestive diseases, posaconazole concentrations were lower compared to those without such conditions (450 ng/mL vs. 1,035 ng/mL), although the difference was not statistically significant (*p* = 0.075) (Lebeaux et al., 2009).

#### 4.14.4 Body weight and BMI

A study by Miceli et al. (2015) observed that patients weighing ≥90 kg or with a BMI ≥30 had lower mean trough concentrations compared to lighter or less obese patients (740 ng/mL vs. 1,320 ng/mL; 890 ng/mL vs. 1,290 ng/mL, respectively).

#### 4.14.5 Type of hematologic malignancies

Posaconazole exposure varied among patients with different hematologic malignancies. HSCT patients exhibited slightly higher concentrations than those with acute myeloid leukemia (AML) or myelodysplastic syndromes (MDS) (1,870 ng/mL vs. 1,440 ng/mL) (Cornely et al., 2016).

#### 4.14.6 Genetic factors

Studies have shown that polymorphisms in the uridine diphosphate-glucuronosyltransferase (UGT)1A4 gene, which metabolizes posaconazole, contribute to variations in drug absorption. Patients with the UGT1A4*3 genotype exhibited lower POS steady-state concentrations compared to those with the wild-type genotype (Suh et al., 2018).

#### 4.14.7 Graft-versus-host disease (GVHD)

Posaconazole concentrations were lower in patients who developed acute GVHD compared to those with chronic GVHD (814 ng/mL vs. 1,413 ng/mL) (Krishna et al., 2007a). Concentrations were also lower in patients who developed gastrointestinal GVHD (1,080 ng/mL vs. 1,420 ng/mL) (Tonini et al., 2012).

## 5 Discussion

Posaconazole is a triazole antifungal agent widely used for the prevention and treatment of various fungal infections. Maintaining therapeutic blood levels is crucial for achieving successful treatment outcomes (Van Daele et al., 2020). This study analyzes the impact of multiple factors on posaconazole blood concentrations, including patient-specific characteristics, drug interactions, formulation differences, and pharmacogenetic variations. Understanding these factors is essential for optimizing posaconazole therapy to ensure both efficacy and safety.

### 5.1 Individual patient factors

Factors such as age, sex, body weight, renal and hepatic function, vomiting, mucositis, and high-fat dietary intake did not significantly influence posaconazole concentrations. However, genetic variations in drug-metabolizing enzymes, such as UGT1A4, were found to affect posaconazole metabolism, highlighting the impact of individual genetic differences on drug concentrations. Diarrhea was found to significantly reduce posaconazole concentrations in this study, and another recent study on population pharmacokinetics also found that diarrhea resulted in underexposure to posaconazole extended-release tablets (Yamada et al., 2024).

### 5.2 Drug interactions

Posaconazole is metabolized partly by liver enzymes, including UGT1A4 and P-glycoprotein (P-gp). The concurrent use of drugs that induce or inhibit these enzymes can significantly alter posaconazole metabolism and blood levels. Co-administration of phenytoin and rifabutin, both inducers of the UGT enzyme system (Anderson, 2004; Reinach et al., 1999), significantly decreased posaconazole concentrations in healthy volunteers using DRTs. However, the specific UGT isoforms induced by these drugs remain unidentified.

### 5.3 Absorption and formulation differences

Absorption plays a crucial role in determining blood concentrations of posaconazole. Drugs such as H2 receptor antagonists (H2A) and PPIs can affect posaconazole absorption by altering gastric pH. Although these drugs did not significantly affect concentrations in DRT formulations, they considerably reduced concentrations in POS formulations. This suggests that DRTs provide more stable posaconazole levels and are less susceptible to variations in gastrointestinal absorption conditions.

### 5.4 Formulation impact

Posaconazole is available in various formulations, each with distinct bioavailability, absorption kinetics, and drug exposure profiles. DRTs achieved significantly higher concentrations than oral suspensions when administered at the recommended doses, suggesting that switching between formulations could influence therapeutic efficacy. Although this study did not compare intravenous formulations with others, intravenous forms are generally designated for treatment-refractory invasive fungal infections, typically in critically ill patients (Sime et al., 2019), with a recommendation to switch to oral administration as soon as clinically feasible.

### 5.5 Dosage effects

The study revealed no significant differences in concentrations between 600 mg and 800 mg daily doses of POS for prophylactic and therapeutic use, respectively. However, for DRTs, concentrations were significantly higher at a 400 mg daily dose compared to 200 mg. In the intravenous form, a 300 mg dose resulted in higher drug concentrations compared to a 200 mg dose. These findings reveal variations in posaconazole concentrations across different formulations and dosing strategies. Oral suspension formulations demonstrated more significant variability compared to DRTs.

### 5.6 Personalized dosing

The results of this study emphasize the importance of individualized administration of posaconazole. Physicians need to make timely adjustments to the dosing regimen based on the patient’s TDM results. Factors such as formulation, dosing regimen, and drug interactions play critical roles in influencing posaconazole exposure, emphasizing the need for individualized approaches in antifungal therapy.

The findings of this research offer a guide for the clinical use of posaconazole in the prevention or treatment of fungal infections in patients with compromised immune systems. This can standardize posaconazole administration, increase treatment efficacy, and lessen adverse effects. Giving immunocompromised individuals DRTs could assist patients in maintaining a more constant level of posaconazole and prevent H2A or PPIs from affecting that concentration. Additionally, patients should refrain from taking medications that interfere with the action of enzymes like P-gp and UGT1A4, which metabolize posaconazole. TDM-based dosing of posaconazole should be a part of posaconazole prophylaxis.

### 5.7 Limitations

This analysis has several limitations. It primarily focused on POS and DRT concentrations rather than intravenous formulations. The lack of randomized controlled trials and the low quality of the included studies could reduce the reliability of the results of this study and lead to publication bias. Pediatric patients require individualized dosing based on body weight. DRT has now been found to have high concentrations in pediatric patients as well (Weerdenburg et al., 2024). However, although we did not exclude pediatric patients from our exclusion criteria, the articles that met the inclusion criteria did not have studies that examined changes in posaconazole concentrations in pediatric patients. This resulted in our inability to explore the factors influencing concentrations after posaconazole administration in pediatric patients. ECMO-related data were not included in this study, which may have led us to omit certain factors affecting concentrations. And finally, this study only focused on the factors affecting the concentration of posaconazole and did not explore the factors affecting the AUC, which may lead to a more one-sided result. However, since there is already a strong correlation between concentration and efficacy, we believe that the efficacy of posaconazole can be judged by exploring the factors that interfere with concentration.

## 6 Conclusion

Posaconazole concentrations exhibit considerable variability depending on the formulation, dosing regimen, and patient population. DRTs provide more stable drug concentrations than oral suspensions and are less susceptible to changes in gastrointestinal absorption conditions. To optimize therapy, patients should avoid medications that affect UGT enzymes whenever possible and carefully monitor posaconazole levels. This is particularly important in cases of diarrhea, which can significantly reduce drug concentrations.

## Data Availability

The original contributions presented in the study are included in the article/Supplementary Material, further inquiries can be directed to the corresponding author.
