# Supplementary material for: Factors affecting posaconazole plasma concentrations: a meta-analysis and systematic review
Source: Front Pharmacol. 2024 Dec 19;15:1450120. doi: 10.3389/fphar.2024.1450120 (PMC11693513; doi:10.3389/fphar.2024.1450120)
Supplement: Supplementary file 1 [file DataSheet1.docx]

| Supplemental Table S1. Basic Information on Posaconazole Concentration Levels | | | | | | | | | | |
| --- | --- | --- | --- | --- | --- | --- | --- | --- | --- | --- |
|  |  |  | Test group | Control group | Test group | | | Control group | | |
|  |  |  |  |  | Mean | SD | N | Mean | SD | N |
| Paul O Gubbins, et al (2006) | Mean ± SD |  | 351.7 ± 165.8 | 478.9 ± 194.2 | 351.70 | 165.80 | 14 | 478.9 | 194.2 | 7 |
| Gopal Krishna, et al (2007) | Mean (% CV) |  | 285 (33) | 513 (53) | 285 | 94.05 | 36 | 513 | 271.89 | 36 |
| R Courtney, et al (2005) | Mean (% CV) | Degrees of Chronic Renal Disease | Mild 631 (47) | 555 (40) | 631 | 296.57 | 6 | 555 | 222 | 6 |
|  |  |  | Moderate 486 (37) | 555 (40) | 486 | 179.82 | 6 | 555 | 222 | 6 |
| Angela Sansone-Parsons (2006) | Mean (% CV) |  | 355 (43) | 121 (75) | 355 | 152.65 | 24 | 121 | 90.75 | 24 |
| Gopal Krishna, et al (2007) | Mean (SD) |  | 776 (769) | 817 (689) | 776 | 769 | 12 | 817 | 689 | 194 |
| Angela Sansone-Parsons (2007) | Mean (% CV) |  | 2607 (40) | 3374 (38) | 2607 | 1042.8 | 24 | 3374 | 1282.12 | 24 |
| G Krishna, et al (2007) | Mean (% CV) |  | 428 (29) | 766 (37) | 428 | 124.12 | 8 | 766 | 283.42 | 12 |
| Gopal Krishna, et al (2007) | Mean ± SD (range) | Sex | 1035 ± 728 (0–3537) | 902 ± 772 (0–3650) | 1035 | 728 | 76 | 902 | 772 | 165 |
|  |  | Age | 888 ± 766 (0–3650) | 994 ± 751 (0–3233) | 888 | 766 | 133 | 994 | 751 | 106 |
|  |  | GVHD status | 814 ± 650 (0–3174) | 1413 ± 842 (0–3650) | 814 | 650 | 158 | 1413 | 842 | 82 |
|  |  | Diarrhea | 609 ± 616 (74–2190) | 989 ± 761 (0–3650) | 609 | 616 | 18 | 989 | 761 | 223 |
| Gopal Krishna, et al (2008) | Mean ± SD | Sex | 556 ± 314 | 602 ± 415 | 556 | 314 | 83 | 602 | 415 | 111 |
|  |  | Age | 627 ± 434 | 18-45 (603 ± 401) | 627 | 434 | 7 | 603 | 401 | 61 |
|  |  |  |  | 45-65 (550 ± 357) | 627 | 434 | 7 | 550 | 357 | 88 |
|  |  |  |  | >65 (615 ± 372) | 627 | 434 | 7 | 615 | ± 372 | 38 |
|  |  | γ-Glutamyl transferase level | 454 ± 262 | 606 ± 388 | 454 | 262 | 32 | 606 | 388 | 149 |
|  |  | Liver enzyme levels | 614 ± 431 | 577 ± 366 | 614 | 431 | 30 | 577 | 366 | 163 |
|  |  | Diarrhea | Mild to moderate (411 ± 235) | 655 ± 398 | 411 | 235 | 55 | 655 | 398 | 137 |
|  |  |  | Severe to life threatening (269 ± 250) |  | 269 | 250 | 2 | 655 | 398 | 137 |
|  |  | Vomiting | Mild to moderate (580 ± 376) | 584 ± 377 | 580 | 376 | 19 | 584 | 377 | 174 |
|  |  | H_2_-receptor antagonist | 579 ± 353 | 583 ± 386 | 579 | 353 | 61 | 583 | 386 | 133 |
|  |  | Proton pump inhibitor | 494 ± 269 | 652 ± 430 | 494 | 269 | 86 | 652 | 430 | 108 |
|  |  | Mucositis | Grades 1–2 (637 ± 381) | 563 ± 374 | 637 | 381 | 66 | 563 | 374 | 123 |
|  |  |  | Grades 3–4 (367 ± 236) |  | 367 | 236 | 3 | 563 | 374 | 123 |
| David Lebeaux (2009) | Median (range) | Prophylaxis-diarrhea | 360 (60-1140) | 840 (30-2500) | 445.4273 | 316.242 | 14 | 960.0717 | 646.7193 | 22 |
|  |  | Prophylaxis-mucositis | 355 (60-480) | 795 (30-2500) | 311.5975 | 163.8639 | 6 | 906.7809 | 605.3087 | 30 |
|  |  | Treatment-diarrhea | 350 (310-510) | 1035 (290-4290) | 385.1472 | 95.3169 | 4 | 1481.7136 | 1171.2667 | 14 |
|  |  | Treatment-digestive diseases | 450 (310-1470) | 1035 (290-4290) | 674.6715 | 452.5764 | 6 | 1515.5014 | 1223.2122 | 12 |
| Allen Moton (2010) | Mean (% CV) | hepatic impairment | 694 (41) | 689 (30) | 694 | 285.54 | 6 | 689 | 206.7 | 6 |
|  |  |  | 724 (15) | 517 (80) | 724 | 108.6 | 6 | 517 | 413.6 | 6 |
|  |  |  | 403 (31) | 608 (35) | 403 | 124.93 | 6 | 608 | 212.8 | 6 |
| Gopal Krishna, etal (2011) | Mean (% CV) | Dosage | 626 (76) | 776 (50) | 776 | 388 | 30 | 626 | 475.76 | 33 |
| Alexander M Bryant, et al (2011) | Mean ± SD | Diarrhea | 0.35 (±0.28) | 0.39 (±0.20) | 350 | 280 | 5 | 390 | 200 | 16 |
|  |  | Vomiting | 0.35 (±0.19) | 0.38 (±0.23) | 350 | 190 | 5 | 380 | 230 | 16 |
|  |  | Mucositis | 0.34 (±0.32) | 0.38 (±0.21) | 340 | 320 | 2 | 380 | 210 | 19 |
|  |  | PPI or H_2_ antagonist | 0.35 (±0.20) | 0.67 (±0.16) | 350 | 200 | 19 | 670 | 160 | 2 |
|  |  | Gender | 0.41 (±0.27) | 0.34 (±0.15) | 410 | 270 | 10 | 340 | 150 | 11 |
| John Ray, et al (2011) | Mean ± SD | Dosage | 306±342 | 137±82 | 306 | 342 | 13 | 137 | 82 | 14 |
| G Krishna, et al (2012) | Mean (% CV) | SD | 1290 (29) | 778 (29) | 1290 | 374.1 | 9 | 778 | 225.62 | 10 |
|  |  | MD | 2940 (46) | 1800 (31) | 2940 | 1352.4 | 8 | 1800 | 558 | 8 |
| J Tonini, et al (2012) | Mean ± SD | Gastrointestinal (GI) GVHD | 1.08±0.72 | 1.42±0.86 | 1080 | 720 | 14 | 1420 | 860 | 15 |
| Ashley L. Ross, et al (2012) | Mean ± SD | Dosage | 0.42±0.32 | 0.36±0.2 | 420 | 320 | 34 | 360 | 200 | 20 |
| Marie-Rose B. S. Crombag (2012) | Median (IQR) | PPI | 0.48 ( 0.22–0.81) | 1.72 (1.14–3.59) | 505.6375 | 494.774 | 12 | 2221.81 | 2463.7944 | 5 |
| Valeria A Bernardo, et al (2013) | Median (range) | Age | 0.8 (0.22-2.04) | 0.6 (<0.125-2.98) | 895.5836 | 481.6064 | 21 | - | - | 12 |
| Piergiorgio Cojutti, et al (2013) | Median (IQR) | PPI | 716 (450.3-1150) | 884.5 (701-1280) | 777.8885 | 593.5135 | 11 | 962.834 | 497.942 | 10 |
|  |  | Dosage | 1550 (1335-1960) | 884.5 (701-1280) | 1622.0525 | 537.5022 | 10 | 962.4582 | 491.131 | 11 |
| W J Heinz (2013) |  | Dosage | mean: 448 (SD: 501 ng/ml) | mean: 525 (median: 412 (range: 12-1634ng/ml,IQR: 508)) | 448 | 501 | 56 | 569.3594 | 496.0126 | 12 |
| Johan Maertens, et al (2014) | Mean (% CV) | Dosage | 1180 (51) | 1430 (42) | 1430 (42) | 600.6 | 24 | 1180 (51) | 601.8 | 21 |
| Wendy M Kersemaekers, et al (2015) | Mean (% CV) | Dosage | 2250 (29) | 2840 (30) | 2840 (30) | 852 | 9 | 2250 (29) | 652.5 | 9 |
| Urshila Durani, et al (2015) | Median (IQR) | Formulation | 1655 (1080–2250) | 798 (572–1500) | 1662.1219 | 907.9598 | 32 | 966.1216 | 704.6104 | 61 |
| Aaron Cumpston, et al (2015) | Mean ± SD | Formulation | 1740 ± 706 | 463 ± 309 | 1740 | 706 | 32 | 463 | 309 | 118 |
| Marisa H Miceli, et al (2015) | Mean ± SD | Diarrhoea | 0.65 ± 0.08 | 1.31 ± 0.13 | 650 | 80 | 5 | 1310 | 130 | 23 |
|  |  | PPI/H2RA | 1.11 ± 0.12 | 1.62 ± 0.32 | 1110 | 120 | 23 | 1620 | 320 | 5 |
|  |  | Body weight (kg) | 0.74 ± 0.09 | 1.32 ± 0.14 | 740 | 90 | 6 | 1320 | 140 | 22 |
|  |  | BMI | 0.89 ± 0.13 | 1.29 ± 0.14 | 890 | 130 | 7 | 1290 | 140 | 21 |
| Kim Vanstraelen, et al (2016) | Median (IQR) | HSCT | 0.26 (0.17-0.43) | 1.08 (0.96-1.38) | 288.4588 | 201.2234 | 34 | 1144.0636 | 325.48 | 33 |
| Oliver A Cornely, et al (2016) | Mean (% CV) | Disease state | 2110 (66) | 1430 (48) | 2110 (66) | 1392.6 | 79 | 1430 (48) | 686.4 | 107 |
| Werner J Heinz, et al (2016) | Mean ± SD | Different nutrition regimens | 1191 ± 673 | 1123 ± 811 | 1191 | 673 | 10 | 1123 | 811 | 17 |
| Aaron N Pham, et al (2016) | Mean ± SD | Formulation | 1.32 ± 0.69 | 0.81 ±0.59 | 1320 | 690 | 86 | 810 | 590 | 176 |
|  |  | Tablet | omeprazole (1.29 ± 0.63) | No acid suppression (1.33 ± 0.61) | 1290 | 630 | 40 | 1330 | 610 | 34 |
|  |  | Suspension | omeprazole (0.69 ± 0.10) | No acid suppression (0.82±0.40) | 690 | 100 | 9 | 820 | 400 | 67 |
|  |  | Tablet | famotidine (1.41 ± 1.06) | No acid suppression (1.33 ± 0.61) | 1410 | 1060 | 12 | 1330 | 610 | 34 |
|  |  | Suspension | famotidine (0.69 ± 0.30) | No acid suppression (0.82 ± 0.40) | 690 | 300 | 100 | 820 | 400 | 67 |
| Hyeon Jeong Suh, et al (2017) | Mean ± SD | Formulation | 1866.5 ± 1278.3 | 1015.6 ± 631.9 | 1866.5 | 1278.3 | 40 | 1015.6 | 631.9 | 174 |
| Morgan Belling, et al (2017) | median (IQR) | Formulation | 1665 (522–3830) | 390 (51–1870) | 2025.8139 | 2508.8665 | 64 | 791.2356 | 1365.2125 | 118 |
| Daniela Stelzer, et al (2017) | Mean ± SD | Formulation | 2509 ± 1495 | 930 ± 682 | 2509 | 1495 | 64 | 930 | 682 | 64 |
| Pierre Peterlin, et al (2018) | Median (range) | HSCT | 1220 (340–3610) | 2400 (550–5390) | 2574.0392 | 1311.1522 | 19 | 1423.4598 | 839.7954 | 24 |
| Hyeon Jeong Suh , et al (2018) | Mean ± SD | gene | 1055.4 ± 634.8 | 884.1 ± 621.2 | 1055.4 | 634.8 | 94 | 884.1 | 621.2 | 36 |
| Manon Launay , et al (2018) | Mean ± SD | PPI | 1.33 ± 1.17 | 1.49 ± 1.07 | 1330 | 1170 | 19 | 1490 | 1070 | 6 |
| Jade M Kozuch , et al (2018) | Mean ± SD | Dosage | 2.39 ± 1.49 | 1.66 ± 0.91 | 2390 | 1490 | 20 | 1660 | 910 | 19 |
| Wirawan Jeong , et al (2018) | Median (IQR) | Formulation | 1.65 (0.97–2.13) | 0.81 (0.48–1.15) | 1579.5 | 876.166 | 78 | 813.5415 | 512.3731 | 47 |
| Tyler K Liebenstein , et al (2018) | Mean ± SD | Formulation | 1296 ± 707 | 788 ± 324 | 1296 | 707 | 40 | 788 | 324 | 30 |
| D Stelzer , et al (2018) | Mean ± SD | prophylaxis | 1893 ± 1235 | 656 ± 580 | 1893 | 1235 | 9 | 656 | 580 | 9 |
|  |  | treatment | 2910 ± 1547 | 959 ± 604 | 2910 | 1547 | 15 | 959 | 604 | 15 |
| Elisabeth Leclerc , et al (2018) | Mean ± SD | Formulation | 1.91 ± 1.06 | 0.82 ± 0.57 | 1910 | 1060 | 50 | 820 | 570 | 104 |
|  |  | diarrhoea | 0.99 ± 0.49 | 1.98 ± 1.0 | 990 | 490 | 6 | 1980 | 1000 | 44 |
| Elodie Gautier-Veyret , et al (2018) | Median (IQR) | Formulation | 1.8 (1.2-2.4) | 1.2 (0.7-1.6) | 1800 | 921.8894 | 41 | 1164.3276 | 701.7666 | 29 |
| Weiyang Li , et al (2020) | Median (IQR) | PPI | 0.55 (0.39–0.75) | 0.70 (0.44–1.32) | 564.1472 | 274.3337 | 53 | 829.3429 | 699.6296 | 21 |
| Tony Lai, et al (2020) | Mean ± SD | PPI | 515.57 ± 436.51 | 850.41 ± 481.52 | 515.57 | 436.51 | 14 | 850.41 | 481.52 | 56 |
|  |  | metoclopramide | 500.11 ± 382.09 | 887.52 ± 486.25 | 500.11 | 382.09 | 18 | 887.52 | 486.25 | 52 |
|  |  | mucositis | 441.33 ± 130.86 | 815.52 ± 498.12 | 441.33 | 130.86 | 6 | 815.52 | 498.12 | 64 |
|  |  | ranitidine | 421.00 ± 12.728 | 794.10 ± 491.94 | 421 | 12.728 | 2 | 794.1 | 491.94 | 68 |
|  |  | enteral feeding | 369.56 ± 127.27 | 906.07 ± 489.31 | 369.56 | 127.27 | 16 | 906.07 | 489.31 | 54 |
|  |  | HSCT | 863.29 ± 519.86 | 569.11 ± 313.99 | 863.29 | 519.86 | 19 | 569.11 | 313.99 | 51 |
| Jihyu Oh, et al (2020) | Mean ± SD | Formulation | 1.631 ± 0.878 | 0.879 ± 0.585 | 1631 | 878 | 154 | 879 | 585 | 88 |
| Hyojin Chae, et al (2020) | Median (IQR) | Formulation | 1308.9 (836.5-1910.8) | mediam 713 (range: 31 - 2962) | 1354.2742 | 798.647 | 513 | 789.9859 | 570.6492 | 122 |
|  |  | GVHD | 1285.1 (878.9-1742.4) | 1416.3 (786.3-2199.5) | 1470.0917 | 1056.4293 | 174 | 1303.0144 | 642.8737 | 339 |


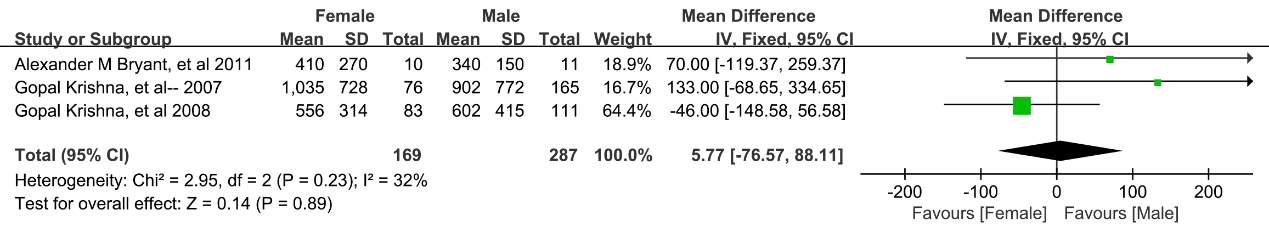


Supplemental Figure S1 Forest plot of the effect of gender on the concentration of posaconazole oral suspension.


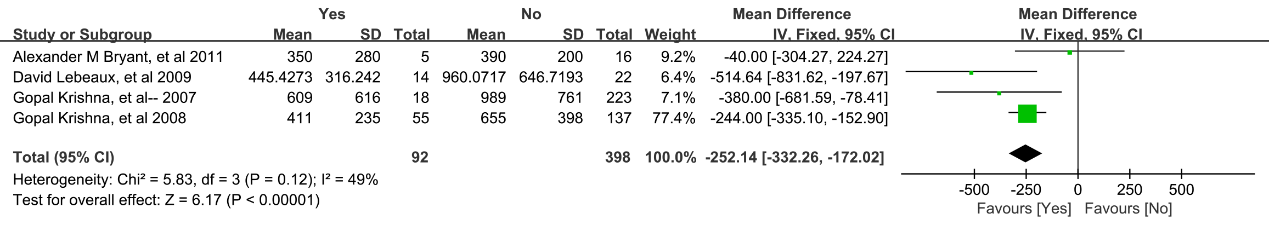


Supplemental Figure S2 Forest plot of the effect of diarrhea on the concentration of posaconazole oral suspension.


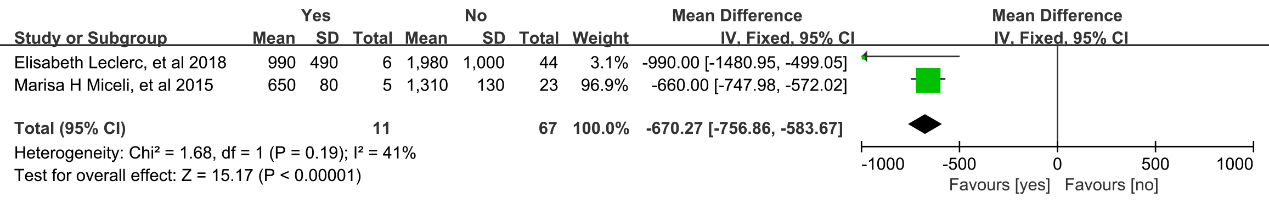


Supplemental Figure S3 Forest plot of the effect of diarrhea on the concentration of posaconazole delayed-release tablets.


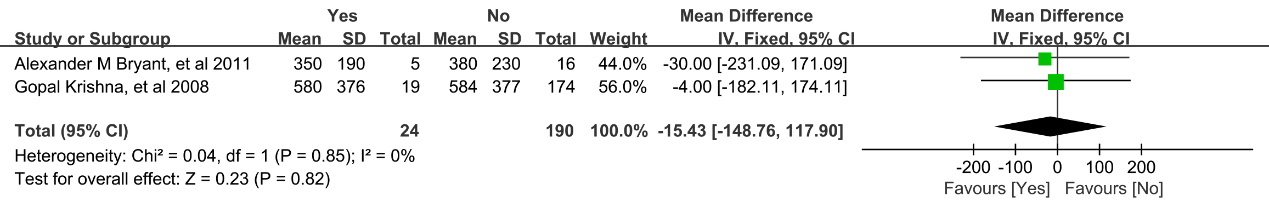


Supplemental Figure S4 Forest plot of the effect of vomiting on the concentration of posaconazole oral suspension.


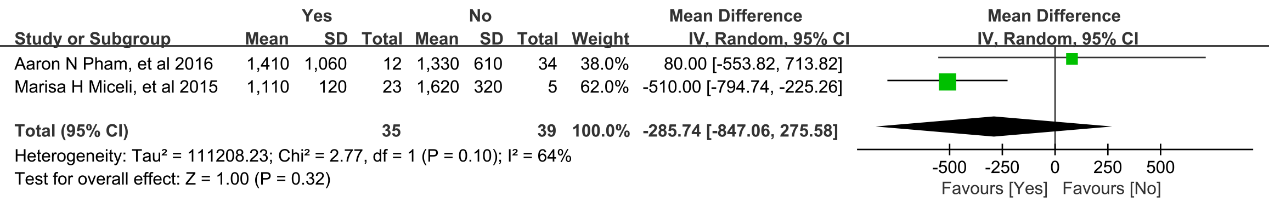


Supplemental Figure S5 Forest plot of the effect of H_2_ receptor antagonists on the concentration of posaconazole delayed-release tablets.


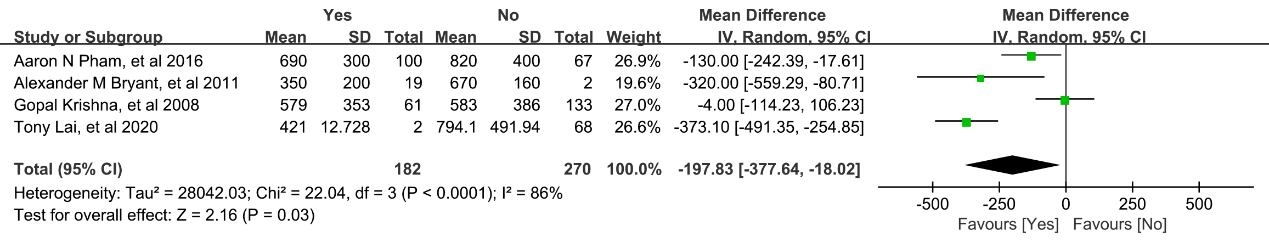


Supplemental Figure S6 Forest plot of the effect of H_2_ receptor antagonists on the concentration of posaconazole oral suspension.


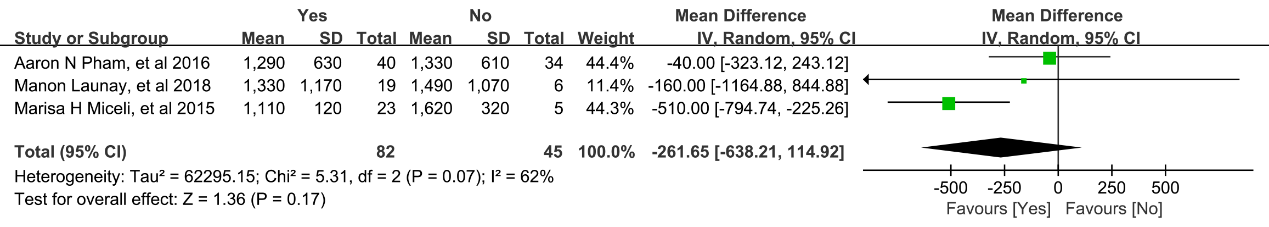


Supplemental Figure S7 Forest plot of the effect of proton pump inhibitors on the concentration of posaconazole delayed release tablets.


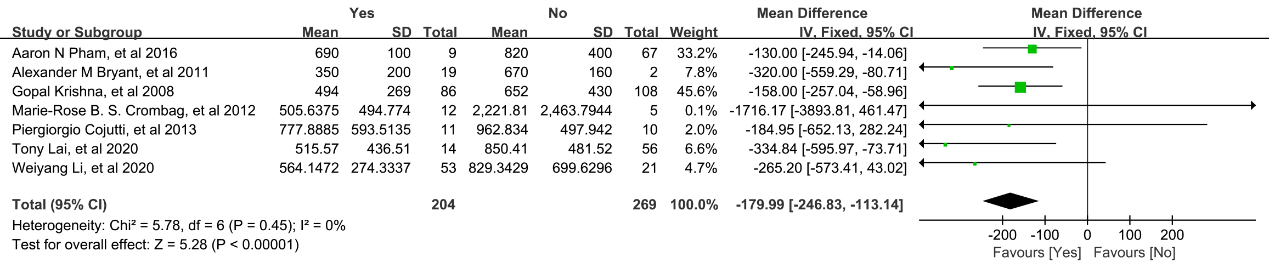


Supplemental Figure S8 Forest plot of the effect of proton pump inhibitors on the concentration of posaconazole oral suspension.


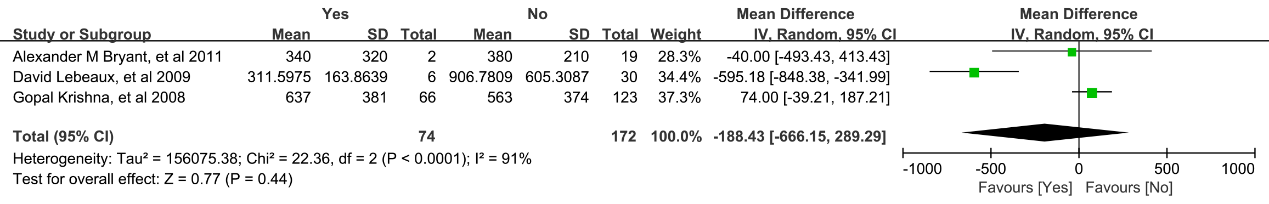


Supplemental Figure S9 Forest plot of the effect of the occurrence of mucositis in patients on the concentration of posaconazole oral suspension.


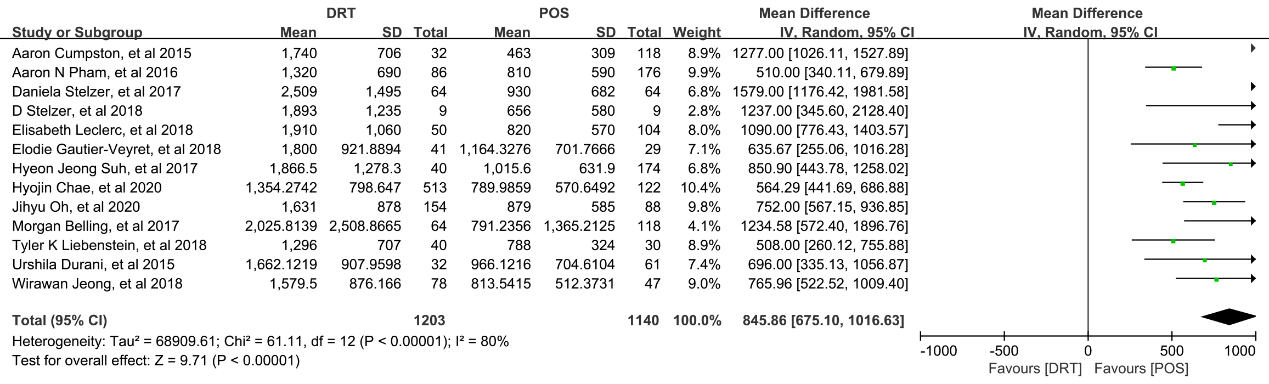


Supplemental Figure S10 Forest plot of the effect of posaconazole formulations on concentration.


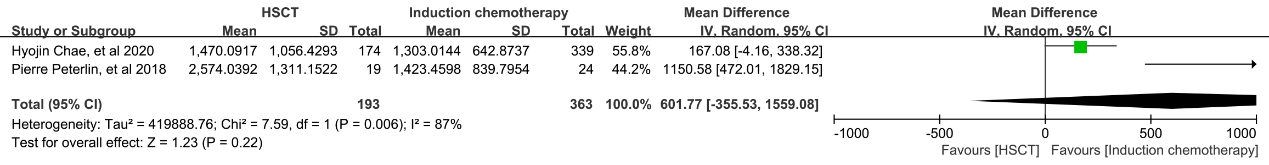


Supplemental Figure S11 Forest plot of the effect of hematopoietic stem cell transplantation and induction chemotherapy on the concentration of posaconazole delayed-release tablets.


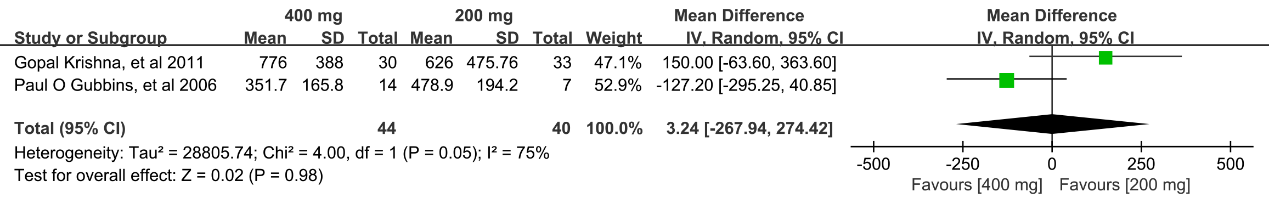


Supplemental Figure S12 Forest plot of posaconazole oral suspension administration of 200mg and 400mg.


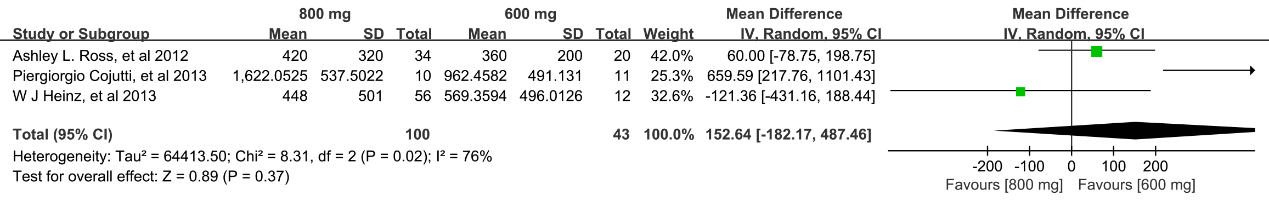


Supplemental Figure S13 Forest plot for posaconazole oral suspension administration of 600mg and 800mg.


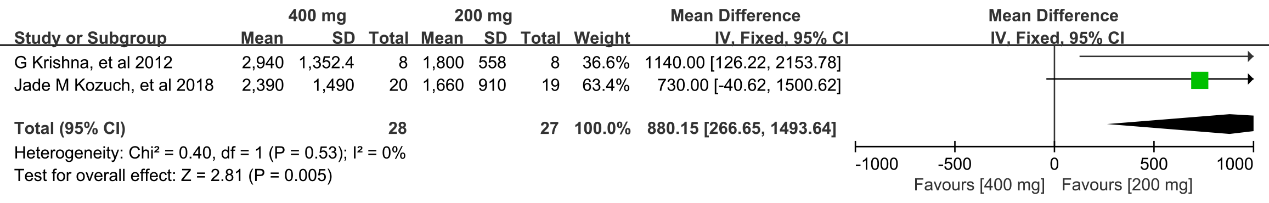


Supplemental Figure S14 Forest plot of delayed-release tablets administered 200mg and 400mg.


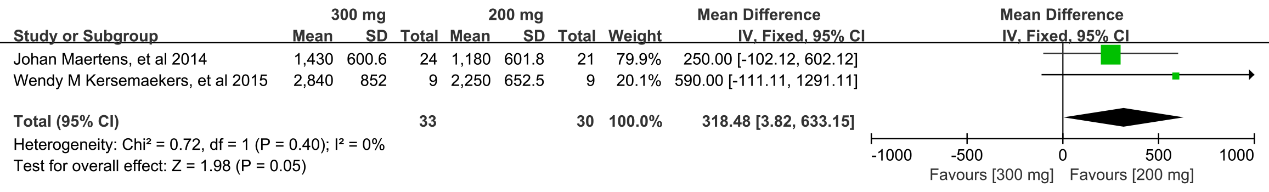


Supplemental Figure S15 Forest plot of intravenous administered 300mg and 200mg.
